# Preparation, Characterization, and *In Vivo* Evaluation of Olanzapine Poly(D,L-lactide-co-glycolide) Microspheres

**DOI:** 10.1155/2013/831381

**Published:** 2013-08-12

**Authors:** Susan D'Souza, Jabar A. Faraj, Stefano Giovagnoli, Patrick P. DeLuca

**Affiliations:** ^1^Sunovion Pharmaceuticals Inc., Marlborough, MA 01752, USA; ^2^Fresenius Kabi USA, Skokie, IL 60077, USA; ^3^Department of Chemistry and Technology of Drugs, Università degli Studi di Perugia, Via del Liceo 1, 06123 Perugia, Italy; ^4^University of Kentucky College of Pharmacy, Lexington, KY 40536, USA

## Abstract

The aim of this study was to prepare injectable depot formulations of Olanzapine using four poly(D,L-lactide-co-glycolide) (PLGA) polymers of varying molecular weight and copolymer composition, and evaluate *in vivo* performance in rats. *In vivo* release profiles from the formulations were governed chiefly by polymer molecular weight and to a lesser extent, copolymer composition. *Formulations A* and *B*, manufactured using low molecular weight PLGA and administered at 10 mg/kg dose, released drug within 15 days. *Formulation C*, prepared from intermediate molecular weight PLGA and administered at 20 mg/kg dose, released drug in 30 days, while *Formulation D*, manufactured using a high molecular weight polymer and administered at 20 mg/kg dose, released drug in 45 days. A simulation of multiple dosing at 7- and 10-day intervals for *Formulations A* and *B* revealed that steady state was achieved within 7–21 days and 10–30 days, respectively. Similarly, simulations at 15-day intervals for *Formulations C* and *D* indicated that steady state levels were reached during days 15–45. Overall, steady state levels for 7-, 10-, or 15-day dosing ranged between 45 and 65 ng/mL for all the formulations, implying that Olanzapine PLGA microspheres can be tailored to treat patients with varying clinical needs.

## 1. Introduction

Schizophrenia is a debilitating lifelong mental illness that is associated with significant and long-lasting health, social, and financial burdens. Worldwide, it affects more than 24 million of the population with treatment costs amounting to several billion dollars annually [1, 2]. Nonadherence to medications that treat schizophrenia type medical illnesses are generally the primary reason behind the below par results offered by the treatment regimens [3]. Additionally, studies describe a wide range in nonadherence rates (24–74%) among patients [4, 5], resulting in frequent hospitalizations, relapse episodes that not only affect the outcome of the treatment but also contribute to its overall cost.

Historically, oral administration of antipsychotics in the form of tablets has been available for the treatment of various schizophrenia type disorders. While being easy to administer, the effectiveness of this type of delivery mechanism faces significant hurdles in most part due to the nature of the illness due to absentmindedness, recoil from ingestion, and so forth. To remedy this, a long-acting injection depot formulation (Haldol Decanoate) was introduced as a treatment option in the 1960s to counter problems facing oral drug delivery [6]. A major advantage to this type of therapy was in the bypassing of the gastrointestinal tract, thereby reducing the amount of medication ingested while minimizing hepatotoxicity and hyperprolactinemia effects [7]. However, the effectiveness of this treatment was hindered due to: (i) prolonged pain arising from the injection site that increased the likelihood of treatment discontinuation, and (ii) irreversibility and inflexibility once administered [8]. 

In recent years, atypical antipsychotics have been gaining popularity and increasingly being used to treat schizophrenia and related disorders. Olanzapine, a thienobenzodiazepine derivative, is one such novel antipsychotic drug and is used in the treatment of schizophrenia and bipolar 1 disorder (a lifelong illness with a variable course) [9]. Olanzapine has been shown to selectively bind to central dopamine D2 and serotonin (5-HT2c) receptors and is effective against the negative symptoms of schizophrenia with a lower incidence of extrapyramidal symptoms. A second generation atypical antipsychotic, Olanzapine is extensively metabolized in liver (1st pass metabolism) by the cytochrome P450 CYP1A2 and CYP2D6 isoenzymes to about 10 metabolites, some of them are inactive while the others cause many adverse effects, followed by glucuronidation [10]. These adverse effects include hypotension, dry mouth, tremors, and somnolence. The drug has a moderate elimination half-life implying that once daily therapy is adequate for treatment of schizophrenic conditions [11]. 

Olanzapine was initially marketed as an oral tablet wherein after administration via the oral route, poor patient compliance was observed along with spitting of the tablet at the time of administration [12]. Later studies attributed poor compliance to the age of the patient population as nearly 20% of all patients with bipolar disorder were adolescents, with a peak age of onset between 15 and 19 years [13, 14]. Administering antipsychotics like Olanzapine via the oral route to the adolescent population is always challenging when compared to adults, especially if Olanzapine is used as the only therapeutic agent to treat the condition in comparison to coadministration with other agents. Such patients may also experience erratic cycles of severe mania and depression or mixed episodes of simultaneous mania and depression. Of particular concern is the misuse of this drug since overdoses are often fatal and occur more frequently with outpatients [15]. For instance, in a 49-week trial involving manic patients, a high percentage (60%) of noncompliers was observed [16]. While such situations require frequent hospitalization and are a recommended standard of medical care, the significant costs involved with inpatient treatment make the utilization of such a nonconducive approach and thus an alternative solution is desirable [11]. 

Research in the area of oral drug delivery was instrumental in the development of an orally disintegrating tablet (ODT) whose design and development was able to offer advantages such as rapid absorption of the therapeutic and ability to bypass the gastrointestinal tract. This approach was utilized by Lilly and led to the development of Olanzapine ODT for the treatment of psychotic episodes [12]. A comparison of Olanzapine ODT with the oral tablet showed improved exposure levels at early time points with the former, with no statistically significant differences in pharmacokinetic parameters between both dosage forms [12]. Despite the ODT being preferred by clinicians, nonadherence to daily oral therapy was once again a major concern in schizophrenic patients, especially in an outpatient setting. Therefore, therapy that increased adherence to medication was believed to be of vital importance in schizophrenic patients suggesting that administration of a long-acting dosage form could reduce the risk of nonadherence to daily oral therapy. Consequently, long-acting injection of Olanzapine (Olanzapine Pamoate for intramuscular administration, dose 10–20 mg) was developed [17] and was found to be bioequivalent to oral Olanzapine therapy [18]. Available as the poorly water-soluble pamoate salt of Olanzapine, prolonged release *in vivo* is achieved by the control of dissolution rate. However, as is commonly noted with most injectable suspensions, drawbacks such as spreading of depot at the injection site and swelling may affect the overall pharmacokinetic profile of the drug. 

Clinical concerns with usage of poorly water-soluble salts as injectable suspensions can be easily addressed by administering dosage forms that contain drug encapsulated in a polymeric vehicle. With the significant advances in the design of polymeric vehicles that can be utilized as delivery matrices, polymers with specific properties can be selected to obtain a desired *in vivo* profile for a given therapeutic agent. The past decade has witnessed significant advancements in the use of polymers like polylactide (PLA) and poly(lactide-co-glycolide) (PLGA) as carriers to deliver drugs for extended periods of time, with minimal to negligible side effects at the site of injection. The PLGA polymer has been approved by the US Food and Drug Administration (FDA) for use in humans and is biodegradable, biocompatible, with low immunogenicity and an extensive safety profile, and is cleared *in vivo* by the Krebs cycle [19]. Biodegradable microspheres formulated using PLGA polymers have been extensively investigated as delivery systems for sustained release of small molecules and biologics [20–22]. In particular, PLGA-based microsphere dosage forms are popular formulations as they provide a means to tailor drug levels *in vivo* for varying duration, from weeks to several months [23–25], thereby reducing the dosing frequency resulting in improved patient compliance [19]. Delivery of drugs like atypical antipsychotics using polymeric carriers, dosed subcutaneously or intramuscularly, is an effective strategy in mitigating patient compliance concerns and related issues as it ensures adherence to therapy leading to improved patient outcomes. This fact is further corroborated by several publications that have emphasized the development and clinical use of long acting dosage forms for the treatment of schizophrenia [26–28]. Thus, the PLGA polymer is an ideal delivery matrix for Olanzapine that could provide initial and sustained levels based on the choice of the polymer used. 

Therefore, the goal of this study was to develop and subsequently investigate the suitability of using PLGA polymers having varying properties like copolymer composition and molecular weight to provide tailored *in vivo* release of an atypical antipsychotic, Olanzapine, via the subcutaneous route.

## 2. Materials and Methods

### 2.1. Materials

Olanzapine (molecular weight 312.44, insoluble in water; sparingly soluble in acetonitrile, and soluble in dichloromethane) was purchased from Cipla Ltd., Bombay, India. PLGA having varying molecular weights (15 and 131 kDa of 75 : 25 PLGA, 30 kDa of 50 : 50 PLGA, 82 kDa of 65 : 35 PLGA) was purchased from Boehringer Ingelheim (Ingelheim, Germany) and Alkermes (Cambridge, MA, USA). All other chemicals were obtained commercially as analytical grade reagents.

### 2.2. Preparation of Microspheres

The four PLGA copolymer ratios and molecular weights evaluated were:

- 15 kDa PLGA, 75 : 25 lactide : glycolide (*Formulation A*),
- 30 kDa PLGA, 50 : 50 lactide : glycolide (*Formulation B*),
- 82 kDa PLGA, 65 : 35 lactide : glycolide (*Formulation C*),
- 131 kDa PLGA, 75 : 25 lactide : glycolide (*Formulation D*).

The microspheres were prepared by a solvent extraction/evaporation method and recovered by filtration [29]. Briefly, a solution of drug and polymer (10–20% polymer concentration) in dichloromethane was injected into an aqueous continuous phase at a ratio between 250 and 350 parts of polymer phase : aqueous phase, under stirring with a Silverson L4R mixer (Silverson machines, MA, USA) at 5000 rpm. Subsequently, the solvents were removed by stirring after which the microspheres were recovered by filtration, suspended in a suitable vehicle, filled into vials, and freeze dried.

### 2.3. Characterization

The microspheres were characterized for mean particle size, surface morphology, bulk density, drug content, and *in vivo* efficacy.

#### 2.3.1. Particle Size

Particle size distribution of the microspheres prior to vialing was determined using a laser diffraction technique (Malvern 2600c Particle Sizer, Malvern, UK). The particles were suspended in 0.05% Tween 80 and counted using a laser sensor. The average particle size was expressed as volume mean diameter in microns (*μ*m).

#### 2.3.2. Surface Morphology

The surface morphology was examined by scanning electron microscopy (SEM) (Hitachi S800, Japan) at an appropriate magnification, after palladium/gold coating of the microsphere sample on an aluminum stub.

#### 2.3.3. Bulk Density

The dry microspheres were quantitatively transferred to a graduated test tube. The test tube was subsequently tapped 50 times from a vertical distance of approximately 0.5 inches and the occupied volume recorded [30]. The tapping process was repeated until the volume occupied by particles remained unchanged. The final volume was recorded as bulk volume, *V*_*b*_, and the tapped bulk density (g/cc) was calculated as *M*/*V*_*b*_, where “*M*” was the weight of microspheres employed.

#### 2.3.4. Drug Content

Olanzapine content in the microspheres was analyzed by a reverse phase HPLC method using an HPLC C-18 column at a flow rate of 1.5 mL/min in a gradient mode. The mobile phases were 0.1% TFA aqueous solution and Acetonitrile with 0.1% TFA. Measurements were performed in triplicate. Drug content (%) was expressed as the “weight of drug in microspheres/weight of microspheres × 100.” Encapsulation efficiency (%) was also calculated for the four formulations. 

#### 2.3.5. *In Vivo* Study

Four groups of male Sprague-Dawley rats (*n* = 6 per group) weighing approximately 300 g were used to evaluate *in vivo* performance of Olanzapine microspheres. The microspheres were injected subcutaneously at the back of the neck (10–20 mg/kg Olanzapine/rat) after reconstitution with water for injection. Blood samples were collected from the tail vein at specific time points. The samples were centrifuged in Microtainer tubes (Becton Dickinson, Franklin Lakes, NJ) and serum was collected. Serum samples were frozen and stored at −20°C until analysis. Serum samples were analyzed at Medtox Laboratories location using a validated method.

#### 2.3.6. Simulation Studies

Multiple dosing pharmacokinetic (MDPK) studies are generally used to direct the selection of an appropriate dosing regimen for a given formulation. However, factors such as expense and labor associated with MDPK studies in animals or human subjects suggest that an alternate strategy may be necessary to elucidate the performance of a dosage form over extended dosing. One such approach is to perform multiple dosing simulations using the plasma concentration-time data from a single dosing regimen. With this approach, individual dosing data is extrapolated to a multiple dosing scenario using the superposition principle. Further, simulation studies also enable selection of a suitable formulation for a multiple dose *in vivo* study.

Previous studies have indicated that Olanzapine follows linear pharmacokinetics after multiple oral dosing. Hence, the plasma concentrations observed after multiple dosing of Olanzapine can be linearly related to the dose and can be predicted from the *C*_max_ and AUC after administration of a single dose [10]. Therefore, this linearity allows simulations of multiple dose pharmacokinetics after continual dosing to be performed using the superposition principle.

In the current study, simulations of serum *in vivo* levels were obtained after subcutaneous administration of *Formulations A* and *B* (single dose at 10 mg/kg), and *Formulations C* and *D* (single dose at 20 mg/kg) were performed using the superposition principle. A 7- and 10-day dosing regimen was used with *Formulations A* and *B* while a 15-day dosing was used with *Formulations C* and *D*. A total of 4 doses were selected for the simulation study as this would be a predictor of steady state concentrations for this molecule.

## 3. Results and Discussion

### 3.1. Characterization of Olanzapine Microspheres 

#### 3.1.1. Particle Shape, Size, and Morphology

**Figure 1:**
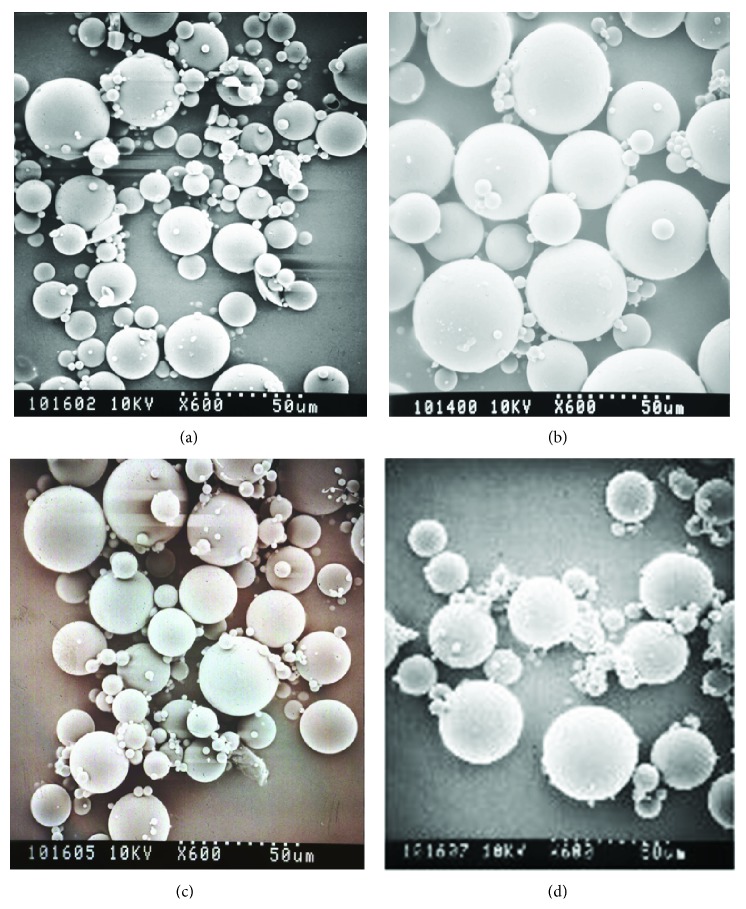
Scanning electron micrographs of Olanzapine PLGA microspheres.

The SEM images of *Formulations A*, *B*, *C*, and *D* are provided in Figure 1. The scanning electron micrographs revealed microspheres having a spherical shape with a smooth nonporous surface and homogeneous particle size distribution. Particle size analysis revealed that *Formulations A*, *B*, *C*, and *D* had a mean volume diameter of 17.0, 16.8, 22.3, and 20.6 *μ*m, respectively (Table 1). The mean volume diameter was similar for *Formulations A* and *B*, both prepared from lower molecular weight PLGA, while the same was true for *Formulations C* and *D*, manufactured using higher molecular weight PLGA. 

The impact of particle size on drug release has been well explored in the field of drug delivery. For instance, a reduction in particle size is a common strategy to enhance dissolution rate of poorly water-soluble drugs [31, 32]. Particle size remains one of the key parameters that affects the degradation rate of the PLGA polymer matrix and thereby drug release rates [33]. Similarly, initial burst also depends on particle size. A reduction in particle size generally depicts an increase in surface area to volume ratio, resulting in a large surface area available for the buffer penetration into the particles and also for a rapid escape of any polymer degradation products. Additionally, with PLGA microsphere dosage forms, the initial burst release phenomenon depends on particle size. In a study published by Yang et al., the authors reported a greater initial burst of a protein drug, bovine serum albumin, from small sized microspheres and attributed it to an increase in surface area [34]. Thus, the initial burst effect depends on two parameters: (a) amount of drug loosely associated with the surface and (b) drug entrapped in the easily accessible porous network. For smaller sized particles, the amount of surface associated drug is expected to be large, and hence, initial burst is not unexpected.

Based on the small particle size of the Olanzapine microspheres, it was inferred that an initial burst would be exhibited by all the formulations evaluated. However, a shorter duration of release was expected for *Formulations A* and *B*, due to lower molecular weight. This suggests that the *in vivo* behavior of Olanzapine from PLGA microspheres could be manipulated to provide varying duration of action.

#### 3.1.2. Bulk Density

Results from bulk density studies are summarized in Table 1. Bulk density values for the formulations varied greatly and were determined to be 0.59, 0.70, 0.60, and 0.96 g/cc for *Formulations A*, *B*, *C*, and *D*, respectively. These data suggest that all four formulations exhibited intermediate to high bulk density values. Between the formulations, a comparison of the data revealed the lowest values for *Formulations A* and *C*, while *Formulation D* exhibited the highest bulk density value, with an intermediate bulk density value for *Formulation B*. 

Assessment of bulk density reveals information on the porous network in the drug loaded microspheres [35]. Thus, any variation in the density or porosity influences the other parameter and hence, impacts drug release behavior. Low bulk density values are a qualitative indicator of the porous network inside the microspheres. Additionally, low bulk density values are also observed with irregular or nonspherical microspheres that display nonoptimal packing [35]. Further, these values can also be correlated with specific surface area and onset of mass loss [36]. Microspheres with high bulk density typically exhibit low values of specific surface area. Conversely, microspheres with a highly porous network will have a low bulk density and thus a faster drug release rate. 

From the bulk density data in the current study, it was inferred that the specific surface area was the lowest for *Formulation D*, but the highest for *Formulations A* and *C*. Generally, low bulk density (high porosity) values in microspheres translate to faster drug release, and hence, certain predictions can be drawn with the bulk density and particle size data: (a) particle size values for the four formulations were similar implying that the impact of this parameter on drug release would be comparable across *Formulations A*–*D*, and (b) due to slightly lower bulk density values for *Formulations A* and *C*, they were expected to show a higher initial burst than *Formulations B* and *D*. 

#### 3.1.3. Drug Content

Results of drug content for Olanzapine PLGA microspheres, as determined by HPLC, are presented in Table 1. *Formulation A* had the lowest drug content (26%) while *Formulation B* had 30% and *Formulations C* and *D* had the highest drug loading (40%). A noteworthy observation was that the encapsulation efficiency was 100% for all the microsphere formulations. These results suggest that the solvent extraction/evaporation method is suitable method for the preparation of Olanzapine microspheres. 

### 3.2. *In Vivo* Studies

#### 3.2.1. Serum Levels of Olanzapine for *Formulations A*, *B*, *C*, and *D*

**Figure 2:**
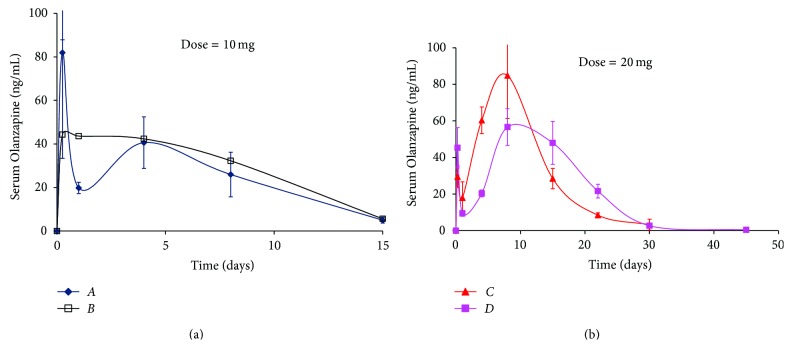
*In vivo* release of Olanzapine PLGA microspheres (*Formulations A* and *B* = 10 mg/kg dose, and *Formulations C* and *D* = 20 mg/kg dose).

Serum levels of Olanzapine for *Formulations A* and *B*, administered at 10 mg/kg dose, and *Formulations C*, and *D*, administered at a 20 mg/kg dose, are indicated in Figure 2. In general, *Formulations A*, *B*, *C*, and *D* describe similar release profiles in that they exhibit an initial burst release followed by a brief trough leading to a secondary peak and a final slow decay phase for the four formulations. 

As predicted, *Formulation A* exhibited the highest initial burst (82 ng/mL) followed by a sharp drop that characterized the trough (20 ng/mL, day 1) leading to a second peak of around 40 ng/mL after which levels exhibited a slow decline through day 15 (Figure 2). In comparison, *Formulation B* exhibited an intermediate initial burst (45 ng/mL) followed by very slight dip in levels (44 ng/mL, day 1) and a secondary peak where values were comparable to the initial burst and trough (43 ng/mL, day 4), with a slow drop in levels till the last time point (day 15). With *Formulations A* and *B* administered at 10 mg/kg dose, the short duration of action (15 days) was expected and attributed to a combination of the properties in the PLGA polymer (copolymer ratio and molecular weight) and microspheres (bulk density and drug content). The high initial burst for *Formulation A* was attributed to a combination of small particle size and low bulk density that allowed for easily accessible drug residing on the surface or in the pores of the microspheres to be released rapidly *in vivo*, while the intermediate burst for *Formulation B* was ascribed to its high bulk density (low porosity).

For *Formulations C* and *D*, administered at 20 mg/kg dose, the duration of action was significantly longer than *Formulations A* and *B* (Figure 2). With *Formulation C*, initial levels were low (29 ng/mL, 6 hours), dropping even lower to reach a trough value of 18 ng/mL by day 1. After the drop in levels by day 1, serum Olanzapine values rose sharply to reach 60 ng/mL by day 4. The true secondary peak level for *Formulation C* was achieved by day 8 (85 ng/mL) after which levels dropped equally sharply to reach about 3 ng/mL by day 30. Unlike *Formulation C* where initial burst was lowest, intermediate burst levels were observed with *Formulation D* (45 ng/mL) that dropped to a stark trough value of 9 ng/mL (day 1). After the trough, serum Olanzapine values began a steady ascent to reach 57 ng/mL (day 8) after which levels once again dropped to reach a final minimum of 3 ng/mL by day 30 (Figure 2). For *Formulations C* and *D*, Olanzapine levels, though monitored through 45 days, were negligible at the last time-point. 

Serum Olanzapine profiles obtained for *Formulations C* and *D* can be explained on the basis of the *in vitro* characterization results. As stated in Section 3.1.3, a low to intermediate burst was expected for *Formulations C* and *D*. Since the bulk density and drug content values were high, a low to intermediate burst implied that the drug remaining in the microspheres would be released in a more sustained fashion. Factoring in the higher lactide content and high polymer molecular weight, the extended duration *in vivo* release was expected for these two formulations. Between *Formulations C* and *D*, the former was manufactured from a 65 : 35 PLGA polymer and hence, faster release of drug to reach a secondary peak was predicted; the *in vivo* results are in agreement with predicted behavior of these polymeric formulations. 

#### 3.2.2. Cumulative AUC for *Formulations A*, *B*, *C*, and *D*

The cumulative area under the curve (AUC), a key pharmacokinetic parameter, for the four formulations, as calculated by the commonly used trapezoidal method (1), is shown in Table 2. Consider

(1) AUC(t1−t2)=[(C1+C2)2]×(t2−t1).

In (1), “*t*” is indicative of time in hours and “*C*” represents “serum concentration of Olanzapine (ng/mL).” Results from AUC calculations indicate that *Formulation A* exhibited the lowest cumulative AUC through 15 days (380 ng × mL/day), with a slight increase in the value for *Formulation B* (449 ng × mL/day). The lower cumulative AUC values for *Formulations A* and *B* were ascribed to the low polymer molecular weights and low drug content for both formulations. A closer examination of the data revealed that despite the high burst with *Formulation A* that contributed about 3% to the cumulative AUC, the net contributions of the time points after the secondary peak were similar to that of *Formulation B*. This was ascribed to the lack of the characteristic peak and trough release profile observed with *Formulation B* (Figure 2) where burst release contributed a meager 1% to the total cumulative AUC. For this reason, the total cumulative AUC value for *Formulation B* was slightly higher than *Formulation A*.

*Formulations C* and *D*, administered at a higher dose (20 mg/kg), demonstrated cumulative AUC values of 1,001 and 932 ng × mL/day through 30 days, respectively, higher than those observed with *Formulations A* and *B* that were administered at 10 mg/kg dose (Table 2). These formulations exhibited low to intermediate initial burst; therefore, the percent of cumulative AUC contributed by this phenomenon was less than 0.4% for *Formulations C* and *D*. A lower amount of initial burst also suggested that the extended duration of PLGA release was due to Olanzapine entrapped in the polymer that was released slowly upon hydrolytic degradation of the 65 : 35 or 75 : 25 lactide : glycolide copolymer. 

In general, analysis of cumulative AUC for *Formulations A–D* revealed the following noteworthy points.

- The contribution of initial burst towards the total AUC for all formulations was minor (equal to or less than 3%).
- Olanzapine was well entrapped in the PLGA polymer matrix and was responsible for over 97% of the cumulative AUC *in vivo*.
- The cumulative AUC obtained with *Formulations C* and *D* was nearly 2 to 3 times greater than that observed with *Formulations A* and *B*, suggesting that selection of an appropriate polymer and microsphere properties would offer tailored release of Olanzapine from PLGA based systems.

#### 3.2.3. Simulation of Multiple Dosing

**Figure 3:**
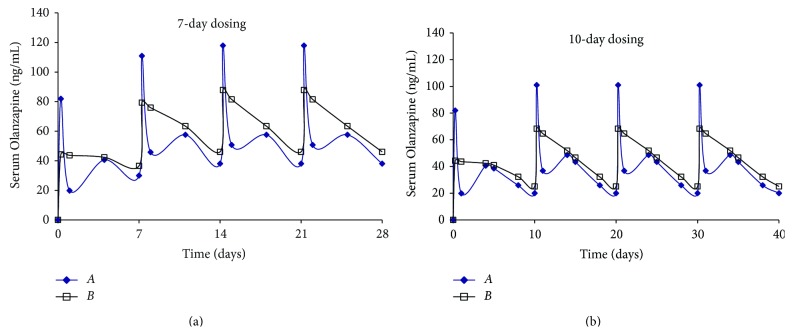
Simulation of multiple dosing regimen (10 mg/kg dose every 7 or 10 days, total = 4 doses) for Olanzapine PLGA microspheres (*Formulations A* and *B*).

Figure 3 shows serum levels for *Formulations A* and *B*, after 4 doses, when administered weekly or once every 10 days. A once weekly and 10-day dosing regimen was selected for *Formulations A* and *B* where the duration of action was short. Once weekly simulation for *Formulation A* revealed that pulsatile behavior was to be expected *in vivo*, similar to what was observed with administration of a single dose. Simulations for doses 2–4 show that levels between 40 and 110 ng/mL are easily achieved with weekly dosing with a slightly lower range for the 10-day dosing. With *Formulation B*, weekly dosing provides serum levels ranging between 50 and 80 ng/mL while 10-day dosing affords slightly lower levels, in a manner similar to that observed with *Formulation A*. The difference between the maximum and minimum serum levels for *Formulation B* was the smallest of all the formulations evaluated. Irrespective of the dosing regimen, Figure 3 indicates that steady state levels are attained between doses 2 and 4 for *Formulations A* and *B*. 

**Figure 4:**
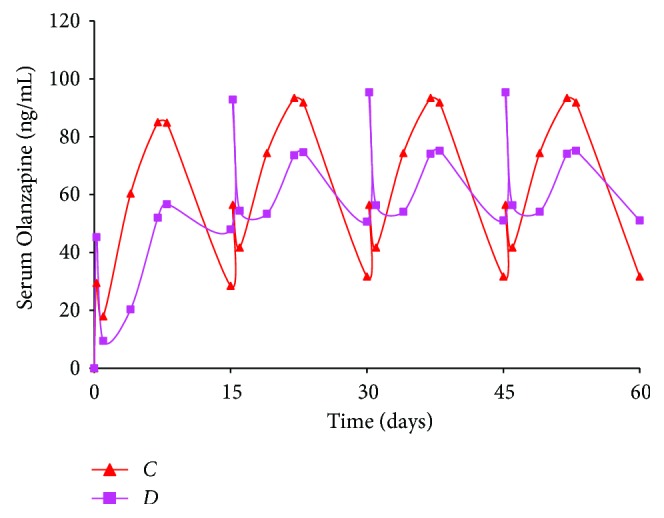
Simulation of multiple dosing regimen (20 mg/kg dose every 15 days, total = 4 doses) for Olanzapine PLGA microspheres (*Formulations C* and *D*).

A 15-day dosing regimen was performed on *Formulations C* and *D*, where the duration of action was considerably longer (Figure 4). The 15-day simulation for *Formulations C* and *D* shows that drug release from the latter formulation was pulsatile. However, serum levels ranged between 30 and 100 ng/mL for both batches through 4 doses. This implies that *Formulations C* and *D*, tailored to release drug for an extended duration, would be excellent candidates for 15-day administration. Such type of therapy has the added benefit of reducing the number of injections required to initiate and maintain adherence to therapy. Overall, simulations for the four formulations suggest that the Olanzapine PLGA microspheres provide a suitable initial burst and maintain release over a period of time *in vivo*.

#### 3.2.4. Steady State Levels

**Figure 5:**
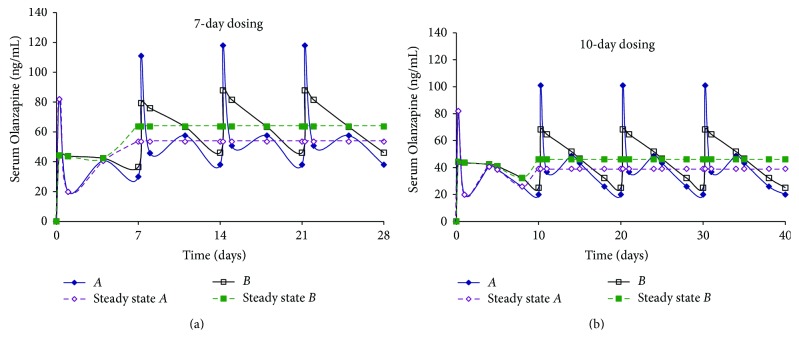
Average steady state concentration for *Formulations A* and *B*.

**Figure 6:**
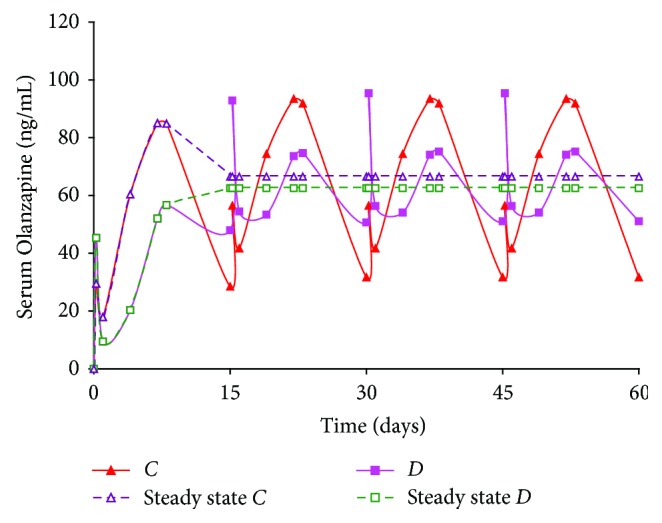
Average steady state concentration for *Formulations C* and *D*.

A comparison of the average steady state concentration for *Formulations A–D* is shown in Figures 5 and 6. The average steady state concentrations were calculated for the four formulations and determined to be 54 and 64 ng/mL for weekly dosing of *Formulations A* and *B*, with slightly lower levels (39 and 46 ng/mL, resp.) for 10-day dosing. A similar calculation for *Formulations C* and *D* (15-day dosing) revealed steady state levels of 67 and 63 ng/mL, respectively. 

Steady state values from the simulation studies provide information on the *in vivo* behavior of the four formulations. For *Formulation A*, dosed weekly, a high burst is expected after which levels drop nearly 30 ng/mL to reach 54 ng/mL and release drug in a sustained fashion through the 4-week dosing interval. Slightly higher and constant steady state levels are expected when *Formulation B* is dosed weekly. As expected, steady state levels for a 10-day dosing regimen are lower for *Formulations A* and *B* (Figure 5). 

For the higher molecular weight longer acting PLGA formulations, higher levels could be achieved with 15-day dosing. In fact, the steady state levels achieved are higher than the initial burst and can be attributed to drug entrapped in the polymeric matrix. 

These results bear strong clinical significance in that drug levels *in vivo* can be tailored to suit patient needs using a systematic scientific approach. Indeed, steady state levels for weekly, 10-day, or 15-day dosing range between 45 and 65 ng/mL, allowing the clinician to utilize a variety of dosage forms for a shorter or longer duration of therapy that is patient specific. Such an approach is highly effective in the treatment of patient populations with schizophrenia and related disorders. 

## 4. Conclusions 

Preparation of injectable depot formulations of an atypical antipsychotic encapsulated within PLGA microspheres is an excellent delivery mechanism that offers the possibility of sustained drug release over a large duration of time. In this study, 4 long-acting formulations of varying molecular weight and copolymer compositions were developed with the intent of illustrating that tailored formulations can provide medical professionals suitable choices in designing therapeutic strategies to treat patients with varying clinical needs. *In vivo* experiments in rats revealed that the Olanzapine formulations would be suitable for weekly, 10-day or, 15-day dosing and would achieve steady state levels by the second dose. The studies and results clearly indicate the utility of the tailored formulation approach to developing long-acting Olanzapine injectable depot preparations. Thus, proper selection of polymer composition and molecular weight will enable customizing drug release from PLGA formulations. Additionally, this strategy depicts a reduction in the frequency of dosing that can prove to be of significant benefit in the development of novel therapy type drugs as we move from animal to human models. 

## Figures and Tables

**Table 1:** Properties of Olanzapine PLGA microspheres.

Formulation	A	B	C	D
PLGA type	75 : 25	50 : 50	65 : 35	75 : 25
Polymer MW	15 kDa	30 kDa	82 kDa	131 kDa
Drug content, %	26	30	40	40
Bulk density, g/mL	0.59	0.70	0.60	0.96
Mean particle size (*μ*m)	17.0	16.8	22.3	20.6
Dose of Olanzapine	10 mg/kg	10 mg/kg	20 mg/kg	20 mg/kg

**Table 2:** Cumulative AUC for Olanzapine PLGA microspheres.

Formulation	*A*	*B*	*C*	*D*
Dose	10 mg/kg	10 mg/kg	20 mg/kg	20 mg/kg
Cumulative AUC (ng × mL/day)	380	449	1,001	932
